# GANT61 and Lithium Chloride Inhibit the Growth of Head and Neck Cancer Cell Lines Through the Regulation of GLI3 Processing by GSK3β

**DOI:** 10.3390/ijms21176410

**Published:** 2020-09-03

**Authors:** Vedran Zubčić, Nikolina Rinčić, Matea Kurtović, Diana Trnski, Vesna Musani, Petar Ozretić, Sonja Levanat, Dinko Leović, Maja Sabol

**Affiliations:** 1 Department of Maxillofacial Surgery, University Hospital Osijek, Ulica Josipa Huttlera 4, Osijek 31000, Croatia; takeshi.zubcic@gmail.com; 2 School of Medicine, The Josip Juraj Strossmayer University of Osijek, Trg Svetog Trojstva 3, Osijek 31000, Croatia; 3 Laboratory for Hereditary Cancer, Division of Molecular Medicine, Ruđer Bošković Institute, Bijenička cesta 54, Zagreb 10000, Croatia; nikolina.rincic@irb.hr (N.R.); matea.kurtovic@irb.hr (M.K.); diana.trnski@irb.hr (D.T.); vmusani@irb.hr (V.M.); pozretic@irb.hr (P.O.); levanat@irb.hr (S.L.); 4 Department of Otorhinolaryngology and Head and Neck Surgery, Maxillofacial Surgery Unit, Clinical Hospital Centre Zagreb, Kišpatićeva ulica 12, Zagreb 10000, Croatia

**Keywords:** hedgehog signaling, head and neck cancer, GLI, GANT61, LiCl, GSK3β

## Abstract

Several signaling pathways are aberrantly activated in head and neck squamous cell carcinoma (HNSCC), including the Hedgehog-Gli (HH-GLI), WNT, EGFR, and NOTCH pathways. The HH-GLI pathway has mostly been investigated in the context of canonical signal transduction and the inhibition of the membrane components of the pathway. In this work we investigated the role of downstream inhibitors GANT61 and lithium chloride (LiCl) on cell viability, wound closure, and colony forming ability of HNSCC cell lines. Five HNSCC cell lines were treated with HH-GLI pathway inhibitors affecting different levels of signal transduction. GANT61 and LiCl reduce the proliferation and colony formation capabilities of HNSCC cell lines, and LiCl has an additional effect on wound closure. The major effector of the HH-GLI signaling pathway in HNSCC is the GLI3 protein, which is expressed in its full-length form and is functionally regulated by GSK3β. LiCl treatment increases the inhibitory Ser9 phosphorylation of the GSK3β protein, leading to increased processing of GLI3 from full-length to repressor form, thus inhibiting HH-GLI pathway activity. Therefore, downstream inhibition of HH-GLI signaling may be a promising therapeutic strategy for HNSCC.

## 1. Introduction

Head and neck squamous cell carcinoma (HNSCC) encompasses tumors arising in the oral cavity, larynx, nasopharynx, oropharynx, hypopharynx, and salivary glands, with an annual incidence of around 880,000 new cases worldwide and around 450,000 deaths [1]. HNSCC has high mortality, since it is usually diagnosed when the disease is locally advanced [2]. Major risk factors are tobacco and alcohol consumption and occupational risks (exposure to wood dust, acid mist, asbestos or solvents in the textile and wood industry) [3]. Infection with human papilloma virus 16 (HPV16) has been determined as an independent risk factor, and the affected individuals are usually younger and non-smokers [4]. Recently, the incidence of tobacco- and alcohol-associated HNSCC has decreased, but HPV-associated HNSCC has increased in developed countries [5]. In Croatia, the majority of HNSCC cases are still HPV-negative and are associated with tobacco and alcohol consumption [6,7]. In HNSCC, cancer stem cells (CSC) are considered to be responsible for tumor initiation, progression, and metastasis, but also for drug resistance and recurrence. Signaling pathways often activated in CSC include the Hedgehog-Gli (HH-GLI), WNT, EGFR, and NOTCH pathways [8]. Treatment of the early stages of HNSCC includes surgery or radiotherapy, while advanced stages are resected surgically and treated with adjuvant radio- or chemoradiotherapy. Immunotherapy has also been implemented for therapy of recurrent or metastatic disease; however, the efficacy is limited due to primary or acquired tumor resistance [9,10]. To bypass potential tumor resistance, other potential molecular targets are being investigated, such as the phosphoinositol 3 kinase (PI3K) pathway, human growth hormone (HGF) pathway, NOTCH signaling pathway, HH-GLI signaling pathway, and angiogenesis regulated by vascular endothelial growth factor (VEGF) signaling [10].

The HH-GLI signaling pathway is a developmental pathway, mostly inactive in the adult organism except in stem cell maintenance and wound healing. The main components are the hedgehog (HH) ligands (Sonic hedgehog, SHH, Indian hedgehog, IHH, and Desert hedgehog, DHH). They bind to the transmembrane receptor patched (PTCH1), which releases its inhibitory effect on the co-receptor smoothened (SMO). This triggers a phosphorylation and ubiquitination cascade in the cytoplasm through several proteins, including beta-transducin repeat containing protein 1 (βTrCP1), glycogen synthase kinase 3 beta (GSK3β), protein kinase A (PKA), casein kinase 1 (CK1), and suppressor of fused (SUFU), which ultimately regulate the processing of glioma-associated oncogene homolog (GLI) proteins. The full-length versions of GLI1-3 proteins are transcriptional activators, while the truncated versions of GLI2-3 are transcriptional repressors. GLI proteins regulate the transcription of many genes involved in proliferation, differentiation, cell cycle regulation, stemness, epithelial-mesenchymal transition (EMT), angiogenesis, and invasiveness, as well as pathway autoregulation through PTCH1 and GLI1 [11].

Most of the studies dealing with the role of the HH-GLI pathway in HNSCC used immunohistochemical staining as the method of choice. In most of them, only GLI1 of the three GLI proteins was stained, and its nuclear localization was associated with metastasis, poor survival, tumor size, and recurrence [12,13,14,15]. GLI1 nuclear expression was also shown to be a predictive biomarker for response to chemoradiation in esophageal cancer [16]. Staining of other proteins of the pathway, namely, PTCH1, SMO, GLI2, and SHH, has also been reported [7,17,18,19,20], and one study demonstrated that HH-GLI pathway proteins show a progressive increase in expression from healthy mucosa, through dysplastic tissue, to carcinoma [21].

In vitro studies on HNSCC cell lines focus mostly on the inhibition of the membrane part of the pathway, protein SMO. Cyclopamine, an SMO inhibitor, can inhibit the growth of esophageal cancer In vitro [22], and it can sensitize HNSCC cells to cisplatin and docetaxel [23]. Vismodegib, another SMO inhibitor, sensitizes HNSCC cells to radiation therapy [24].

Almost all studies examine the role of GLI1 as the main marker of the HH-GLI pathway activity, with only a few examining the roles of GLI2 and GLI3. HNSCC spheroid cultures demonstrate EMT, CSC-like phenotype, and upregulation of *GLI1* and *GLI2* genes [25]. Rodrigues et al. recently demonstrated that GLI3 is important in the CSC population of oral squamous cell carcinoma (OSCC) and is involved in cell proliferation, invasion, and stemness of these cells [26]. It is known that GLI proteins can be activated by non-canonical signaling and can bypass this upstream inhibition. Therefore, we decided to investigate downstream inhibitors in several HNSCC cell lines. We focused our research on two inhibitors, a direct GLI inhibitor GANT61, and lithium chloride (LiCl), a GSK3β inhibitor. GSK3β can have a stimulatory or inhibitory effect on GLI proteins, depending on its phosphorylation status. In non-stimulated cells, GSK3β (phosphorylated at Tyr216) is constitutively active and phosphorylates a range of targets to keep them in an off-state [27]. LiCl promotes phosphorylation of GSK3β at the Ser9 position, leading to the phosphorylation of GLI proteins and their processing into repressor forms and/or degradation [28].

## 2. Results

### 2.1. The HH-GLI Signaling Pathway Is Active in HNSCC Cell Lines

**Figure 1:**
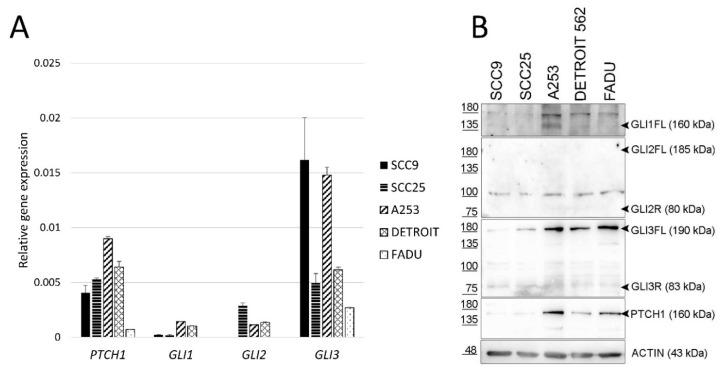
Gene and protein expression of HH-GLI pathway components in HNSCC. (**A**) Relative gene expression of *PTCH1*, *GLI1*, *GLI2,* and *GLI3* determined by quantitative Real-Time Polymerase Chain Reaction (qRT-PCR). Gene expression is shown relative to the level of the housekeeping gene *RPLP0*. (**B**) Protein expression determined by Western blot. FL refers to the full-length protein (activator form), while R refers to the repressor form.

HH-GLI signaling pathway genes *PTCH1*, *GLI1*, *GLI2,* and *GLI3* are expressed in all analyzed HNSCC cell lines. Out of the three *GLI* genes, *GLI3* shows the strongest expression in all analyzed cell lines (Figure 1A). The same expression pattern is visible at the protein level. The full-length GLI3 protein (GLI3FL) shows the strongest expression of all GLI proteins (Figure 1B). The calculated mass of the GLI1 protein is 118 kDa [29], However, the full-length size of GLI1 has been shown to migrate to 160 kDa [30], and we detected a signal at this size only in the A253 line, while it is very faint in other lines. For GLI2, we could not detect the protein in its full-length form of 185 kDa nor the repressor form at 80 kDa, but only a non-specific band around 100 kDa. The PTCH1 protein was detected in all cell lines, in some more strongly than others (Figure 1B). Therefore, we can conclude that the HH-GLI signaling pathway is active in all studied HNSCC cell lines.

### 2.2. HNSCC Cell Lines Respond to Downstream Inhibition More Efficiently Than to Upstream Inhibition

To determine if the HH-GLI pathway activity can be modified in these lines, the cell lines were treated with three different HH-GLI pathway inhibitors: cyclopamine (1.25–10 mM), GANT61 (5–25 μM), and lithium chloride (LiCl) (5–40 mM). Cyclopamine acts on the membrane part of the HH-GLI pathway, LiCl modifies the activity of a cytoplasmatic regulatory kinase GSK3β, while GANT61 inhibits GLI proteins. In four out of five cell lines, GANT61 has the strongest inhibitory effect on cell proliferation, followed by LiCl, while cyclopamine shows the weakest or no effect (Supplementary Figure S1). The only exception is the hypopharyngeal squamous cell carcinoma cell line FaDu, which is the most responsive to LiCl inhibition, followed by cyclopamine, while GANT61 shows the weakest effect. Median lethal dose (LD50) values were determined for all tested cell lines using the Prism 8 program (GraphPad Software, San Diego, CA, USA), and the summary results are presented in Table 1. Based on these results, 5 and 10 μM GANT61 and 10 and 20 mM LiCl were used in the subsequent experiments for all cell lines. Cyclopamine was not tested further as its effects were very weak for these cell lines compared to GANT61 and LiCl. The doses of cyclopamine that would be required to achieve the LD50 values were close to the concentrations that induce the non-specific toxicity independent of the HH-GLI pathway [31].

**Figure 2:**
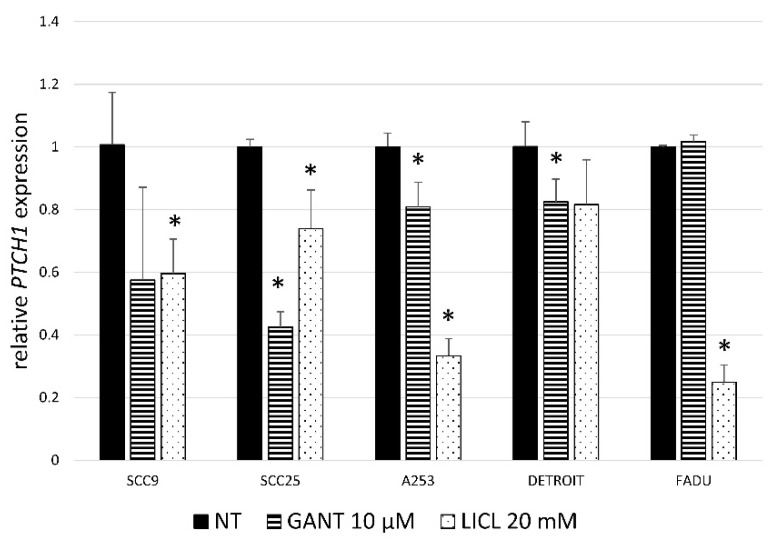
Relative gene expression of *PTCH1*. *PTCH1*, the target gene of the HH-GLI signaling pathway, 24h after GANT61 or LiCl treatment. LiCl downregulates *PTCH1* expression in 4/5 HNSCC cell lines, and GANT61 in 3/5 lines. * denotes a statistically significant difference from non-treated (NT) cells (*p* < 0.05).

To check if the pathway activity is functionally inhibited by these treatments, target gene *PTCH1* expression was determined with qRT-PCR. GANT61 treatment significantly downregulates the expression of the *PTCH1* gene in three HNSCC cell lines (SCC9, SCC25, A253). LiCl treatment downregulates the expression of *PTCH1* in all lines except Detroit562 (Figure 2).

### 2.3. GANT61 and LiCl Regulate GLI3 Protein Levels in HNSCC

**Figure 3:**
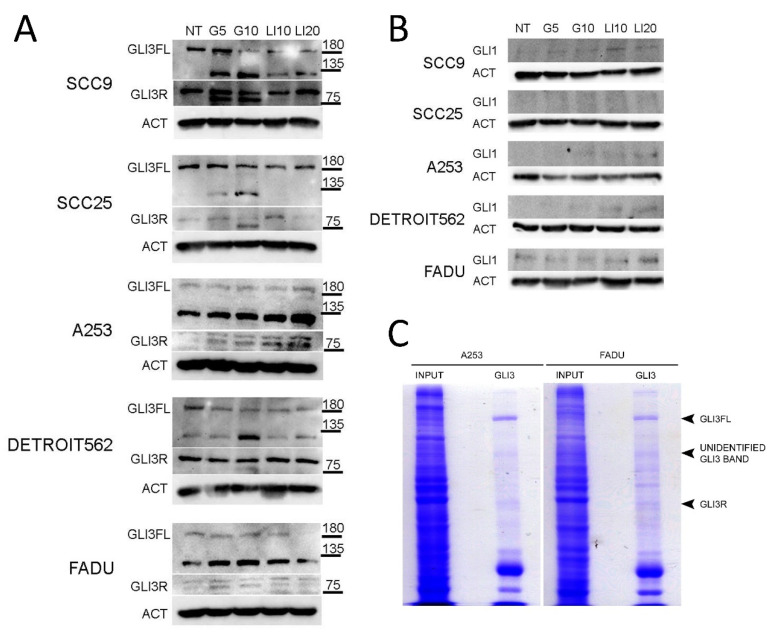
The effect of GANT61 and LiCl treatment on the expression of GLI proteins. (**A**) GLI3 and (**B**) GLI1 protein expression after treatment with HH-GLI pathway inhibitors GANT61 and LiCl (G5—5 μM GANT61, G10—10 μM GANT61, LI10—10 mM LiCl, LI20—20 mM LiCl). (**C**) Coomassie gel stain of immunoprecipitation with GLI3 antibody (AF-3690, R&D) for two HNSCC cell lines treated with GANT61. In both cell lines, the IP successfully detects the GLI3FL and GLI3R bands. The unidentified GLI3 band is also faintly detected in both tested cell lines.

The levels of GLI1 and GLI3 expression were determined after inhibition with GANT61 or LiCl to determine the effect of inhibition on the balance of GLI activators and repressors. The GLI3 protein was the most consistently expressed of all three GLI proteins. To our knowledge, there are no reported isoforms of GLI3 in the literature apart from the full length (GLI3FL) at 190 kDa, and the repressor form (GLI3R) at 83 kDa. The GLI3 protein was present in the full-length form in all examined cell lines, suggesting it acts as the pathway activator in HNSCC cell lines. GLI3R was found strongly expressed in two HNSCC lines, and weakly expressed in three HNSCC lines. In SCC9, SCC25, and Detroit562 cell lines, GLI3FL was downregulated after GANT61 and LiCl treatment. Interestingly, an additional, yet unidentified band around 120 kDa was detected in all five HNSCC cell lines after GANT61 treatment. In the untreated cells, the band is not detected in two lines, weakly detected in one, and strongly in two lines. Upon GANT61 treatment, it is upregulated in all cell lines, and in the case of SCC9 and A253 by LiCl treatment as well (Figure 3A). The GLI1 protein was continuously poorly expressed and barely detectable in all tested HNSCC cell lines (Figure 3B). To determine if the unidentified GLI3 band was specific, we performed immunoprecipitation of GANT61-treated lines A253 and FaDu with a different GLI3 antibody (AF-3690, R&D) followed by direct gel staining with Coomassie. This specific antibody is not validated for Western blot application, only for immunoprecipitation, so to avoid possible non-specific staining during Western blot, the detection of the proteins was done directly in the gel. The band was not as strong as in the Western blot, but a faint line of the unidentified GLI3 band could be detected in both cell lines (Figure 3C). Immunoprecipitation also detected GLI3FL very clearly, and GLI3R faintly, corresponding to the Western blot results.

### 2.4. The Effect of LiCl Inhibition is Mediated by GLI3 Processing by GSK3β

**Figure 4:**
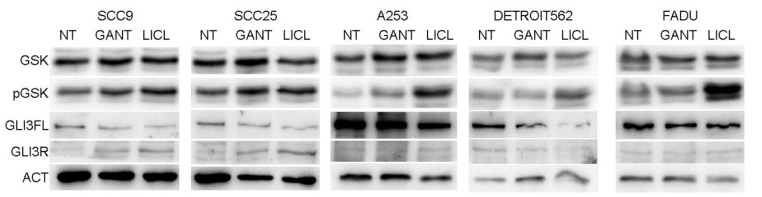
The role of GSK3β phosphorylation in GLI3 protein activity. Western blot showing phosphorylation of GSK3β (pGSK) compared to total GSK levels in non-treated (NT) or GANT61 (10 μM) or LiCL (20 mM) treatment. All HNSCC cell lines show an increase in GSK3β phosphorylation with LiCl, and three of the lines also with GANT61 treatment. This effect is closely followed by the downregulation of GLI3FL.

To test the effect of LiCl on the HH-GLI signaling pathway, phosphorylation of GSK3β was investigated by Western blot. It is known that LiCl treatment increases the inhibitory Ser9 phosphorylation of GSK3β. In all HNSCC cell lines, LiCl increased the Ser9 phosphorylated form of the GSK3β protein, while the total GSK3β levels remained unchanged. This phosphorylation affects GSK3β activity, leading to increased processing of GLI3 from full length to repressor form. This effect can be seen as reduced GLI3FL levels in all HNSCC cell lines, but the increase of GLI3R is detected only in some lines (Figure 4). GANT61 treatment acts directly on the level of the GLI3 protein, also shifting the balance of activator/repressor forms toward the repressor, thus inhibiting pathway activity.

### 2.5. Downstream Inhibition Affects Colony Forming and Wound Closure Capabilities of HNSCC Cell Lines

**Figure 5:**
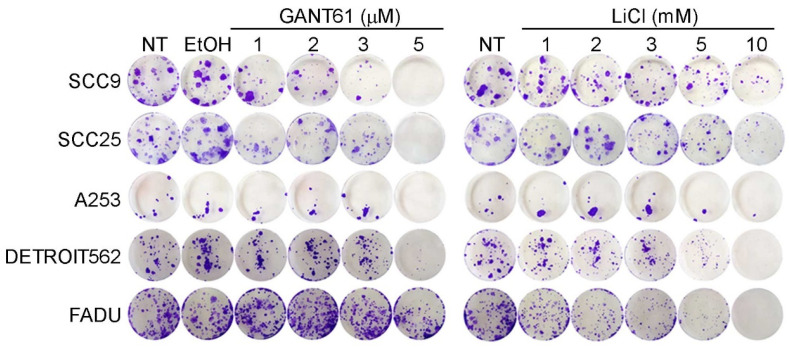
Colony forming assay on HNSCC cell lines. All cell lines show a dose-dependent decrease in colony forming ability with both GANT61 and LiCl. EtOH – vehicle control.

To test the colony forming ability of HNSCC cell lines after GANT61 or LiCl treatment, 1000 cells were plated in a 6-well and maintained for 14 days in medium containing different concentrations of GANT61 and LiCl. All the cell lines showed a dose-dependent decrease in colony forming ability after treatment with either compound. In most cell lines GANT61 was more effective, but in Detroit562 and FaDu lines, LiCl was more effective (Figure 5).

**Figure 6:**
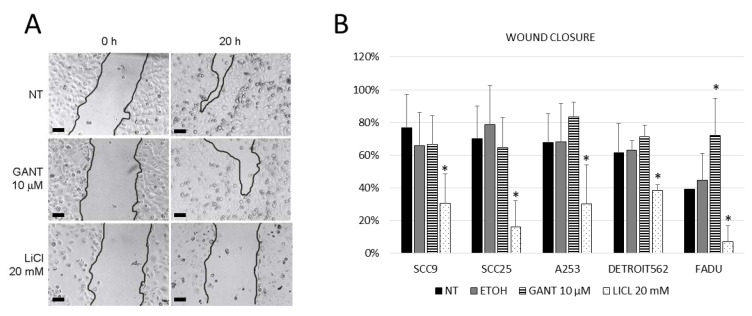
The wound healing assay. (**A**) Digital photographs of wound healing processes (scale bar=200µm). (**B**) Quantification of wound closure. GANT61 inhibition has almost no effect on the wound closure compared to the non-treated cells (NT), with the exception of a slight increase in wound closure for the Detroit562 and FaDu cell lines. LiCl treatment inhibits wound closure capability in all cell lines. EtOH—vehicle control. * denotes statistically significant difference from non-treated (NT) cells (*p* < 0.05).

Wound healing assay was used to examine the proliferative and migratory ability of cells (Figure 6A). In all tested HNSCC cell lines, wound closure was inhibited by LiCl, while GANT61 treatment slightly enhanced wound closure only in Detroit562 and FaDu cells (Figure 6B). As both compounds inhibit the proliferation of these cells, but the wound closure is inhibited only with LiCl, it is likely that this effect is due to the change in the migratory ability of cells, but this would need to be confirmed by other assays, such as the Transwell assay. This suggests that in HNSCC cell lines, GLI proteins generally do not affect the migration of cells. Migratory ability of cells is not regulated by the HH-GLI signaling pathway, but rather by a different GSK3β-triggered mechanism, since LiCl treatment affects the migratory potential of these cells.

## 3. Discussion

In this work we demonstrate for the first time the effect of downstream HH-GLI pathway inhibitors GANT61 and LiCl on HNSCC cell lines. It has been demonstrated previously that the HH-GLI signaling pathway is upregulated and activated in HNSCC [12,13,14,18,20]. The majority of these studies, however, focused only on the GLI1 transcription factor, with few of them examining GLI2, and none of them testing for GLI3 staining. Similarly, in vitro studies on HNSCC cell lines focused mostly on SMO inhibition, with no studies examining potential non-canonical downstream activation of GLI transcription factors and downstream inhibition.

In our set of five HNSCC cell lines, the most uniformly expressed GLI protein is GLI3, while GLI2 is undetectable, and GLI1 is poorly detectable at both mRNA and protein levels. In this study we have demonstrated by the MTT assay that GANT61 and LiCl show much stronger growth inhibition of HNSCC cells than previously used cyclopamine. Different cell lines respond slightly differently, with most of them showing stronger sensitivity to GANT61 and only one line, FaDu, to LiCl. The colony forming ability of HNSCC cell lines is reduced after GANT61 or LiCl treatment, but the wound closure is affected only by LiCl and not by GANT61 treatment. Since LiCl regulates GSK3β activity, and this protein regulates many other targets except GLI (e.g., β-catenin, p53, AP-2, NF-κB) [27], it is likely that the effect on wound closure is mediated by a different mechanism and not by GLI proteins. Both GANT61 and LiCl treatment downregulate the levels of the GLI3FL protein and upregulate the GLI3R form, suggesting proteolytic processing and inhibition of the HH-GLI pathway through GLI3 activator/repressor balance. GLI1 remained mostly unaffected by either treatment, again confirming GLI3 as the pathway regulator. Our findings are supported by a recent paper by Rodrigues et al., who demonstrated a crucial role of GLI3 in cell proliferation and invasion of the OSCC cancer stem cell (CD44high) population [26]. To determine the effect of LiCl on GLI3 levels, the ratios of GSK3β and Ser9 phosphorylated GSK3β (pGSK3β) were examined after GANT61 and LiCl treatment. LiCl generally increases GSK3β phosphorylation, resulting in downregulation of GLI3FL and upregulation of GLI3R. This mechanism has been demonstrated previously in colon cancer cell lines, and it occurs in tumors where the GSK3β activator (Tyr216 pGSK3β) and inhibitor (Ser9 pGSK3β) are in disbalance, with Tyr216 pGSK3β acting as an oncogene [28]. Recently, Nayak et al. showed the effect of nanoquinacrine (NQC) on GSK3β in CSC isolated from the SCC25 line: upon NQC treatment, total GSK3β levels are increased, while pGSK3β levels are decreased, as are GLI1 levels [32]. The effect we detected for LiCl treatments seems to work in the opposite manner: LiCl leads to phosphorylation of GSK3β at Ser9, leading to increased processing of GLI3FL to GLIR. The difference observed between the two studies may be due to the pre-selection of the CSC population in the case of the NQC study. This may have selected for cells with different levels of GSK3β and GLI proteins than the levels we observed in the unselected population of the same cell line. In the CSC population, the Ser9 pGSK3β is far more expressed than the total GSK3β, while in this case the distribution is the opposite. Interestingly, GANT61 treatment, and in some cases LiCl treatment, induced an unidentified GLI3 band on Western blots. We could confirm this by immunoprecipitation with an IP-specific antibody followed by Coomassie stain, but the intensity of the band was not sufficient for fragment identification by mass spectrometry. Therefore, we cannot speculate on the nature or role of the detected GLI3 band, but only demonstrate its upregulation after GANT61 treatment. Taken together, all these results show that the HH-GLI signaling pathway is affected by inhibitors acting downstream of SMO. The major pathway effector in these cell lines was shown to be GLI3, which is present in the full-length form in proliferating cells, but is processed into the repressor form when cells are treated with GANT61 or LiCl. This suggests that downstream pathway inhibitors may be more effective in potential future clinical use for treatment of HNSCC, especially in combination with other signaling pathways often found upregulated in HNSCC.

## 4. Materials and Methods

### 4.1. Cell Culture and Cell Assays

HNSSC cell lines SCC9, SCC25, A253, Detroit562, and FaDu were purchased from the ATCC (no. TCP-1012, Manassas, VA, USA) and maintained in the recommended media. A-253 was maintained in McCoy’s 5A medium (Merck KGaA, Darmstadt, Germany), SCC9 and SCC25 in DMEM:HAM’S=1:1 (Dulbecco’s Modified Eagle Medium: Ham’s F12) medium (Merck KGaA, Darmstadt, Germany), and FaDu and Detroit562 in EMEM (Eagle’s Minimum Essential Medium) medium (Merck KGaA, Darmstadt, Germany), all supplemented with 10% FBS (Fetal Bovine Serum, Merck KGaA, Darmstadt, Germany). The cell lines are derived from different anatomical sites: SCC9 and SCC25 originate from the oral cavity (tongue), FaDu from the hypopharynx, Detroit562 from the metastatic site (pleural effusion of pharyngeal cancer), and A253 from the salivary gland. All the cell lines are established and frequently used models of HNSCC, and were purchased as an HNSCC panel designed by the ATCC. According to the available data, all these lines are HPV-negative.

For MTT (3-(4,5-Dimethylthiazol-2-yl)-2,5-Diphenyltetrazolium Bromide) assays, cells were plated at 2 × 10^3^ cells/well in 96-well plates and treated with a range of concentrations of different HH-GLI pathway inhibitors: cyclopamine 1.25–10 µM (Selleck Chemicals, Houston, TX, USA), GANT61 5–25 μM (Selleck Chemicals, Houston, TX, USA), and LiCl 5–40 mM (Kemika, Zagreb, Croatia) for 24–72 h, and cell viability was measured on LabsSystems Multiskan MS microplate reader (Thermo Fisher Scientific, Waltham, MA, USA) after incubation with MTT. The treatment was done in quadruplicate for each dose, and the experiment was repeated twice.

For colony forming assay, 1000 cells/well were plated in a 6-well plate, left to attach for 24 h, and then treated with 1–5 μM GANT61 or 1–10 mM LiCl. Cells were kept in culture for 2 weeks to allow for colony formation, with media with compounds changed twice per week. Cells were then washed with PBS, fixed with 4% paraformaldehyde, and stained with crystal violet to visualize the colonies. The experiments were performed in triplicate.

For wound healing assay, 10^5^ cells/well were plated in 24-well plates and left for 24 h to attach. Two scratches per well were made with a 10 μL pipette tip; cells were washed with PBS (Phosphate Buffered Saline) to remove floating cells and were treated with GANT61 or LiCl. Images of the scratch were taken immediately after washing with PBS and 24 h later at the same location. Eight images were taken for each treatment, and the images were processed using the MRI Wound Healing Tool plugin for FIJI [33] to calculate the wound area. The experiments were done in triplicate.

For gene/protein extraction, cells were treated with 5 and 10 μM GANT61, or 10 and 20 mM LiCl for 24 h. Cells were collected by scraping with the cell scraper, washing in PBS, and collecting the cell pellet, which was used for either RNA or protein extraction.

### 4.2. Quantitative Real-Time Polymerase Chain Reaction (qRT-PCR)

RNA was extracted from cell pellets with TRIzol reagent (Invitrogen, Carlsbad, CA, USA) per the manufacturer’s instructions. One microgram of RNA was reverse transcribed into cDNA using the High Capacity cDNA synthesis kit (Thermo Fisher Scientific, Waltham, MA, USA), and qRT-PCR was performed on the CFX-96 instrument (Bio-Rad Laboratories, Hercules, CA, USA) using SsoAdvanced SYBR Green (Bio-Rad Laboratories, Hercules, CA, USA). The expression of *PTCH1*, *GLI1*, *GLI2,* and *GLI3* genes was measured as described previously [7], and the fold change was calculated relative to the *RPLP0* housekeeping gene.

### 4.3. Western Blotting

Total proteins were extracted by sonication in RIPA (Radioimmunoprecipitation assay buffer) buffer containing protease and phosphatase inhibitors, and the protein concentration was measured using the BCA (Bicinchonic Acid) kit (Thermo Fisher Scientific, Waltham, MA, USA); 40 μg of protein was loaded on 7% PAA (Polyacrylamide) gel. After electrophoresis, they were transferred to a nitrocellulose membrane (Amersham BioSciences, Little Chalfont, England, UK), blocked with 5% milk and incubated with primary antibodies overnight. Antibodies used for detection were as follows: rabbit anti-GLI1 (Cell Signaling Technology, V812, 1:200, Danvers, MA, USA), mouse anti-GLI2 (Santa Cruz Biotechnology, sc-271786, 1:200, Dallas, TX, USA), rabbit anti-GLI3 (GeneTex GTX104362, 1:1000, Irvine, CA, USA). Actin (60008-1-Ig, ProteinTech, 1:4000, Rosemont, IL, USA) was used as a loading control. After washing in TBST (Tris-Buffered Saline, 0.1% Tween^®^ 20 Detergent), secondary antibodies HRP (Horseradish Peroxidase)-conjugated anti-rabbit (BD Pharmingen, 554021, 1:6000, San Jose, CA, USA) and anti-mouse (BD Pharmingen, 554002, 1:8000, San Jose, CA, USA) were applied for 1h at room temperature, washed, and visualized using SuperWest Signal Pico and Femto reagents (Thermo Fisher Scientific, Waltham, MA, USA) on Uvitec Image Alliance 4.7 instrument (UVItec, Cambridge, England, UK).

### 4.4. Immunoprecipitation and Coomassie Staining

For immunoprecipitation (IP), proteins were extracted from GANT61-treated FaDu and A253 cell lines using TENN buffer (50 mM Tris, 5 mM EDTA, 150 mM NaCl, 0.5% NP-40, pH 8.0) supplemented with protease inhibitors (Roche, Basel, Switzerland). Protein concentration was determined using the Pierce BCA Protein Assay Kit (Thermo Fisher Scientific, Waltham, MA, USA). Immunoprecipitation was performed using Protein G-coated Dynabeads (Life Technologies, Carlsbad, CA, USA) according to the manufacturer’s instructions (Invitrogen, Rev. 005, Carlsbad, CA, USA). For Gli3 immunoprecipitation, 1000 μg of proteins and 5 μg of Gli3 antibody (AF-3690, R&D Systems, Minneapolis, MN, USA) were used for each IP sample. Samples were incubated with the Dynabead–antibody complex overnight at +4 °C. Samples were eluted with 1× loading buffer and heated for 5 min at 95 °C before electrophoresis in 7% PAA gel. After gel electrophoresis, the proteins were fixed in 50% (*v/v*) ethanol and 10% (*v/v*) acetic acid for 1 h at room temperature followed by further fixation in 50% (*v/v*) methanol and 10% (*v/v*) acetic acid overnight at +4 °C. The gels were stained in Coomassie Blue R250 (LKB Bromma, Bromma, Sweden) staining solution (3g/L Coomassie Blue R250, 45% (*v/v*) methanol, 10% (*v/v*) acetic acid in water) for 4 h with gentle agitation, and afterwards destained in 50% (*v/v*) methanol and 10% (*v/v*) acetic acid. The destaining solution was changed several times until the protein bands were completely visible. The gels were stored in 5% (*v/v*) acetic acid at +4 °C.

### 4.5. Statistical Analysis

Normality of data distribution was tested using the D’Agostino-Pearson test. An independent samples t-test was used for comparing wound healing and gene expression between non-treated and treated cells. Two-tailed *p* values < 0.05 were considered statistically significant. Statistical analysis was performed using MedCalc v19.1.6 (MedCalc Software Ltd., Ostend, Belgium).

## 5. Conclusions

GANT61 and LiCl, downstream HH-GLI pathway inhibitors, inhibit the proliferation and colony forming capability of HNSCC cells. The upstream inhibitor cyclopamine, on the other hand, requires high doses to produce an effect on HNSCC cell lines. This suggests that the downstream components of HH-GLI signaling are activated at least partly non-canonically in HNSCC. Wound closure of HNSCC cells is not affected by GANT61 but it is reduced after LiCl treatment, suggesting that the effect of LiCl on wound closure is mediated through another pathway independent of HH-GLI. The main effector of HH-GLI signaling in HNSCC is the GLI3 protein, which is the most expressed of all three GLI proteins and is responsive to GANT61 and LiCl inhibition. LiCl increases the inhibitory Ser9 phosphorylation of GSK3β, leading to increased processing of GLI3 from the full-length form to the repressor form, and inhibiting the pathway. Therefore, downstream inhibition of HH-GLI signaling in HNSCC may be a promising therapeutic strategy.

## Figures and Tables

**Table 1:** Median lethal dose (LD50). LD50 values for each of the tested compounds.

Line	Tissue	Cyclopamine (µM)	LiCl (mM)	GANT61 (μM)
SCC9	Tongue	>10	11.45	>25
SCC25	Tongue	9.51	15.24	6.83
A253	Salivary gland	6.48	14.56	8.36
DETROIT562	Pharynx	>10	11.4	9.54
FADU	Hypopharynx	>10	5.53	19.7

## Supplementary Materials

The following are available online at https://www.mdpi.com/1422-0067/21/17/6410/s1, Figure S1: Results of the MTT assay.

## Abbreviations

HH-GLI	Hedgehog-Gli
LiCl	Lithium chloride
HNSCC	Head and neck squamous cell carcinoma
HPV16	Human papilloma virus 16
CSC	Cancer stem cells
EGFR	Epidermal growth factor receptor
PI3K	Phosphoinositol 3 kinase
VEGF	Vascular endothelial growth factor
PTCH1	Patched 1
HH	Hedgehog
SMO	Smoothened
βTrCP1	Beta-transducin repeat containing protein 1
GSK3β	Glycogen synthase 3 beta
PKA	Protein kinase A
SUFU	Suppressor of fused
CK1	Casein kinase 1
GLI	Glioma-associated oncogene homolog
EMT	Epithelial-mesenchymal transition
NQC	Nanoquinacrine
DMEM:HAM’S	Dulbecco’s Modified Eagle Medium: Ham’s F12
EMEM	Eagle’s Minimum Essential Medium
FBS	Fetal bovine Serum
MTT	3-(4,5-Dimethylthiazol-2-yl)-2,5-Diphenyltetrazolium Bromide
PBS	Phosphate Buffered Saline
RIPA	Radioimmunoprecipitation assay buffer
BCA	Bicinchonic Acid
PAA	Polyacrylamide
TBST	Tris-Buffered Saline, 0.1% Tween® 20 Detergent
HRP	Horseradish Peroxidase
TENN	50 mM Tris, 5 mM EDTA, 150 mM NaCl, 0.5% NP-40, pH 8.0
