# Supplementary figures and images for: GANT61 and Lithium Chloride Inhibit the Growth of Head and Neck Cancer Cell Lines Through the Regulation of GLI3 Processing by GSK3β
Source: Int J Mol Sci. 2020 Sep 3;21(17):6410. doi: 10.3390/ijms21176410 (PMC7504345; doi:10.3390/ijms21176410)

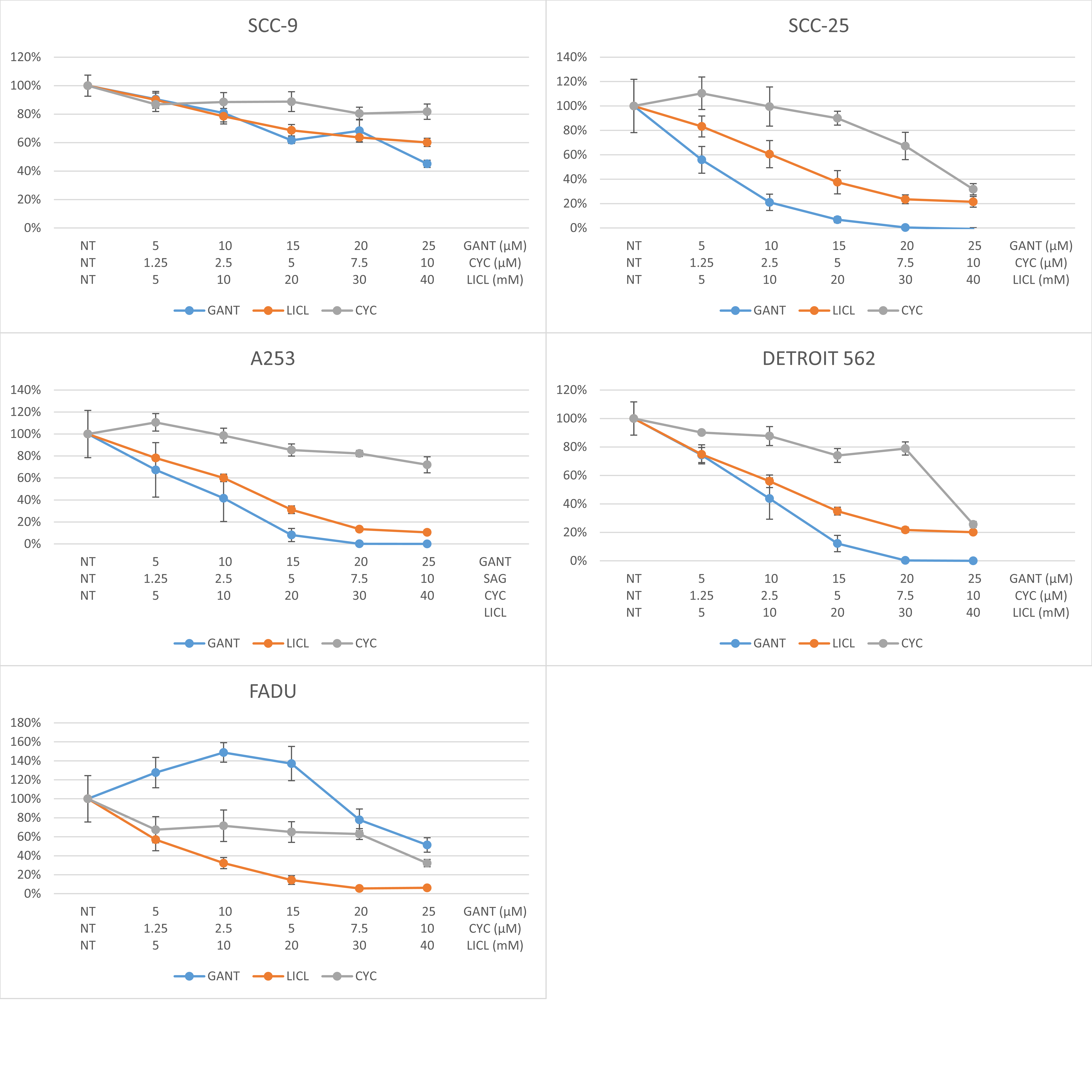

Supplement: Supplementary file 1 [file ijms-21-06410-s001.jpg]
